# The Quest for System-Theoretical Medicine in the COVID-19 Era

**DOI:** 10.3389/fmed.2021.640974

**Published:** 2021-03-29

**Authors:** Felix Tretter, Olaf Wolkenhauer, Michael Meyer-Hermann, Johannes W. Dietrich, Sara Green, James Marcum, Wolfram Weckwerth

**Affiliations:** ^1^Bertalanffy Center for the Study of Systems Science, Vienna, Austria; ^2^Department of Systems Biology & Bioinformatics, University of Rostock, Rostock, Germany; ^3^Department of Systems Immunology and Braunschweig Integrated Centre of Systems Biology, Helmholtz Centre for Infection Research, Braunschweig, Germany; ^4^Endocrine Research, Medical Hospital I, Bergmannsheil University Hospitals, Ruhr University of Bochum, Bochum, Germany; ^5^Ruhr Center for Rare Diseases (CeSER), Ruhr University of Bochum, Witten/Herdecke University, Bochum, Germany; ^6^Section for History and Philosophy of Science, Department of Science Education, University of Copenhagen, Copenhagen, Denmark; ^7^Department of Philosophy, Baylor University, Waco, TX, United States; ^8^Molecular Systems Biology (MOSYS), University of Vienna, Vienna, Austria; ^9^Vienna Metabolomics Center (VIME), University of Vienna, Vienna, Austria

**Keywords:** Organismal Systems Medicine, systems theory, dynamic equilibrium, polypharmacology, multi organ disease, excess mortality rate, multi-level view model

## Abstract

Precision medicine and molecular systems medicine (MSM) are highly utilized and successful approaches to improve understanding, diagnosis, and treatment of many diseases from bench-to-bedside. Especially in the COVID-19 pandemic, molecular techniques and biotechnological innovation have proven to be of utmost importance for rapid developments in disease diagnostics and treatment, including DNA and RNA sequencing technology, treatment with drugs and natural products and vaccine development. The COVID-19 crisis, however, has also demonstrated the need for systemic thinking and transdisciplinarity and the limits of MSM: the neglect of the bio-psycho-social systemic nature of humans and their context as the object of individual therapeutic and population-oriented interventions. COVID-19 illustrates how a medical problem requires a transdisciplinary approach in epidemiology, pathology, internal medicine, public health, environmental medicine, and socio-economic modeling. Regarding the need for conceptual integration of these different kinds of knowledge we suggest the application of general system theory (GST). This approach endorses an organism-centered view on health and disease, which according to Ludwig von Bertalanffy who was the founder of GST, we call Organismal Systems Medicine (OSM). We argue that systems science offers wider applications in the field of pathology and can contribute to an integrative systems medicine by (i) integration of evidence across functional and structural differentially scaled subsystems, (ii) conceptualization of complex multilevel systems, and (iii) suggesting mechanisms and non-linear relationships underlying the observed phenomena. We underline these points with a proposal on multi-level systems pathology including neurophysiology, endocrinology, immune system, genetics, and general metabolism. An integration of these areas is necessary to understand excess mortality rates and polypharmacological treatments. In the pandemic era this multi-level systems pathology is most important to assess potential vaccines, their effectiveness, short-, and long-time adverse effects. We further argue that these conceptual frameworks are not only valid in the COVID-19 era but also important to be integrated in a medicinal curriculum.

## The Complexity of COVID-19 Quests for an Integrative Framework With a Focus On a Bio-Psycho-Social Model

**Figure 1:**
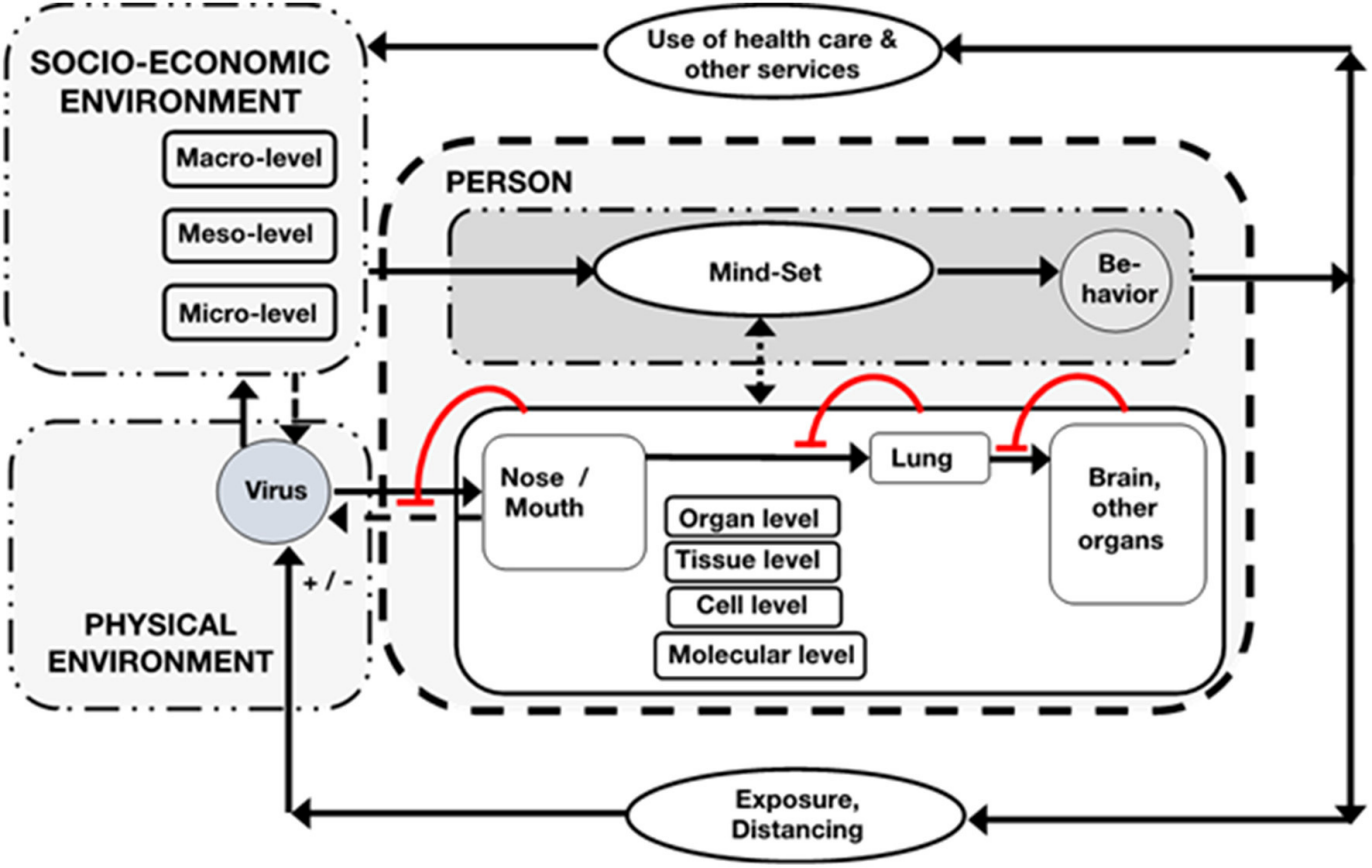
Systemic compartment model of the individual person with its respiratory system that primarily is confronted with and affected by SARS-CoV-2, that invades cells within tissues of respiratory (and others) organs of the organism, step by step. But invasion can be attenuated by local defense mechanisms (circular lines with transoms). Spreading of the virus can occur (stippled arrow). The person can experience the sickness by symptoms (stippled double arrow) and/or obtains and utilizes the information provided by the respective socio-economic system on micro-, meso-, and macro-level and its knowledge about the virus and its prevention and treatment. With this mindset, depending on social context, the person might change behavior by reduction of the exposure (–) by lockdown, distancing, quarantine etc. or will exert risky behavior (+) with respective consequences for the social environment. A major feedback loop is also organization of health care and other services (6, 7) as well as access to these health care services which is not equally distributed in the society (5, 8).

Molecular systems medicine and computational medicine were helpful for the understanding and management of COVID-19 pandemic. Moreover, the importance of mathematical modeling in epidemiology demonstrates the benefits of generic system models that can be used to compare causally different cases (such as the Spanish flu, Ebola and Covid-19) and are also necessary to lead political decisions (1, 2). The societal importance of integrated medical knowledge has become particularly obvious during the COVID-19 pandemic. The understanding and management of COVID-19 pandemic exposes the dissociation of specialized diversity of medicine such as virology, epidemiology, public health, internal medicine, etc. Molecular analysis of mechanisms of infection, epidemiological data on spreading and their mathematical extrapolation alone are insufficient to foresee and avoid catastrophic developments. This exemplifies that empirical research on Covid-19 necessitates analysis of systemic feedback and feedforward mechanisms as well as collateral effects on all levels of organismic organization, on the “ecology of the person” (3) and on the level of institutional management of the pandemic. For example, for differential understanding of the high rate of case fatalities on the population level clinical knowledge of individual courses of the disease must be integrated with views of basic research in various disciplines not only of immunology, but also endocrinology and even neurobiology. Regarding adherence to prevention regulations, psychology of distancing, and social sciences of lock downs must be considered in order to depict the real-life situation of people (3, 4). Higher mortality rates among some population groups cannot be understood through molecular, or even “biological” factors alone, but also involves consideration of psycho-social conditions of life, socioeconomic disparities, and sociocultural orientations; COVID-19 is a syndemic (5). An integration of all these factors into a comprehensive conceptual framework for COVID-19 is proposed in Figure 1. A general system-theoretical bio-psycho-social model for medicine and the education of health professionals has been suggested already in the 1970 by George Engel (9). Regarding this (nearly forgotten) integrative and multidimensional model a *bio-psycho-social pathology* on individual and population level could improve understanding varieties, for example in clinical Covid-19 courses and also could have practical effects in management of the pandemic. But it also would enable a wider understanding of societal conflicts between *restrictive hygiene suggestions, rights for freedom*, and *impaired economic vitality* (see Figure 1). These conflicts exert a strong disturbance on everyday life organization and need a comparative and sophisticated discussion. Smart health care delivery can lower thresholds for access and improve acceptance of the targeted population. In case of in-patient treatment units also a human-centered management structure and style, e.g., “systemic management concepts,” could prevent burn-out of the staff and could enhance success of public health goals (6, 7, 10). Later on, we sketch those issues focusing on somatic processes but being aware of environmental factors as contexts. In the context of human sciences the term “environment” has two epistemic meanings: “subjective” environment according to Jacob von Uexküll (11), and “objective” environment in the sense of Ernst Haeckel (12). This difference corresponds to the clinical data, e.g., if someone is clinically obese and subjectively not aware of it. Integration of data across such different domains is a key task which is exactly the domain of system theory that aims to transform bio-psycho-social data sets to a framework of a functional language that represents an ontology of functions as well as their quantification as discussed later. Furthermore, the need for the conceptual integration of different organismal subsystems to understand the mechanisms of the pandemic raises basic questions about the epistemic power of contemporary systems medicine, which is discussed below. Regarding the multi-organ manifestation of COVID-19, a co-evolution of new disease ontologies, data integration and interoperability strategies that use omics-based classifications and combine them with clinical ontologies are highly fruitful (13).

## From Molecular to Organismal Systems Medicine

Medicine today relies on three major pillars: (i) clinical knowledge and practical experience, (ii) classical diagnostics and evidence-based treatment on the basis of expected value decision making and (iii) multiomics combined with advanced statistical/mathematical analysis such as machine learning and mathematical modeling. Here, precision medicine and molecular systems medicine (MSM) are highly utilized and successful approaches to improve understanding, diagnosis and treatment of many diseases, based on data from multiomics technologies, data statistics and modeling (14–21). Genome-wide-association studies (GWAS) of COVID-19 are rather at the beginning but are highly promising to reveal critical illness cases (22, 23). For data gathering, diagnostics and prediction models machine and deep learning techniques and applications of artificial intelligence are of utmost importance but also need to be critically reviewed (24–29). Altogether, there is a risk that classical medical knowledge, especially qualitative, and intuitive knowledge of organismic pathology, will be lost before the transition to MSM can be implemented clinically. Also, these technologies neglect the bio-psycho-social dimension of medicine as discussed above (4). Interestingly, the technology- and data-driven epistemology of MSM is not yet sufficiently understood regarding the gap between correlation and causation [(30); Table 1 and discussion below], especially comparing it with the very special bedside-epistemology of physician-based and patient-based observations and experiences of health and disease. The combination of implicit biochemical reductionism of physiology and data-reductionism of health phenomena implicates the lack of conceptual inter-level and interdisciplinary integration, namely the neglect of the epistemic weight of clinical experience that is concerned with the whole person. Current documentation within electronic health records (EHRs) is not designed to treat the patient as an organism, but rather as a suite of documentation regarding the clinical encounter and for billing purposes (13). We can learn a lot from the rare disease community (among others) where there is a need to support multi-species and multi-modal data integration to inform diagnostics and treatment discovery. Most of this documentation regarding the patient happens outside the EHRs in order to support the observations of the patient as an organism (13). We have not well-applied this approach to COVID patients yet. In consequence, not only a unidirectional but rather a bidirectional relation between bench and bed (physician and patient) could improve efficacy of translational medicine. This could be “vertical transdisciplinarity” that complements “horizontal transdisciplinarity” as conventional interdisciplinarity and that combines scientific knowledge production with physicians and patients observations and experiences (49–52).

**Table 1:** Epistemological desiderata of systems thinking in biology.

**Problem**	**Literature**
Bilateral relations between data and theory, difficulties of causal inference based on correlations	(31, 32)
Reduction and holism, whole–parts relations: can the knowledge of molecular biology explain higher functions of the whole organism?	(33)
Limits of bottom-up explanations of social phenomena by molecular biology	(34)
Systemic multilevel ontologies and emergence	(35)
Chance and necessity: Is there a significant difference between randomness and determinism?	(36)
Examples of top-down causation	(37)
Epistemology of computational modeling	(38)
The meaning of terms like “information,” “function,” and “structure”	(39)
Scaling problems	(40–42)
The structure of explanations and theories in biology	(43)
The ontology of life	(44)
The limited explanatory power of evolutionary theory	(45, 46)
The concept of goal-directedness as teleonomic but not teleological property of living systems	(47)
The relevance of the concept of self-organization	(48)

*These topics are dealing with conceptual problems that underly the need for systems medicine*.

In consequence, we take a conceptual and theoretical approach that is *organism-centered* conceiving the *organism as system of organs, tissues*, and *cells* and that also envisions the organism as a *living system-in-the-world*. We appeal to the need for an “Organismal Systems Medicine” (OSM) in the sense of Bertalanffy (53, 54) to complement MSM by accounting for the *systemic* and *ecological context* of the organism.

This approach can be seen as a complementary procedure to the bottom-up methodology of current MSM as it is an organism-centered (or: person-centered) top-down functional analysis. This holistic starting point of biomedical research is aware of contextual factors such a psycho-social factors that come up as risk factors and/ or protective factors for health and disease. One of the central concepts of OSM is the adaptive, self-organized dynamic equilibrium (“flow equilibrium”) of partially antagonistically operating components of the system. *Dynamic equilibrium* is constituted by the assumption of hierarchical partial *antagonisms* and *synergisms* between *activators* and *inhibitors* that converge on operators of different organizational levels of complex organisms (organs, tissue, cells, and molecules). This is a heuristically fruitful concept to organize observed phenomena and data and it is also a guiding principle for organizing experimental and field research as we will show later.

## Epistemology of Molecular and Organismal Systems Medicine

Altogether, a differentiated but integrated systemic methodology could improve our understanding and managing of COVID-19 but also future challenges. Accordingly, it has to be considered that epistemic limitations of valuable MSM show up focally, but they are not worked out in a broader way. In contrast, systems biologists and philosophers alluded to methodological difficulties/limitations of claims of early systems biology (55, 56). Some examples for important metatheoretical topics that would frame methodology of systems medicine are listed here [(30); see Table 1]: The part-whole problem, bottom-up vs. top-down causation, mechanistic vs. nomological explanations, complexity reduction, epistemics of interdisciplinarity, determinism and self-organization, correlation and causation, emergence vs. reductionism, robustness vs. homeostasis. Several of these problems are relevant for a holistic MSM (57). Selected aspects are discussed in the following.

### Functional Organization of the Organism

A major challenge in biomedical research is to understand the *functional organization* of a living system across multiple levels. Several taxonomies were presented in history of systems science that intend to capture conceptually a limited set of “essential functions” like respiration, circulation, reproduction, or more general like adaptation, assimilation, integration, differentiation, etc. (58). Biological functions (e.g., defense functions such as inflammation) can be localized at different levels of organization (e.g., molecules, cells, tissue, organs), using different methodologies. Biases sometimes occur as a result of downgrading factors that are left out of the analysis when focus is directed at a specific functional level. This type of bias was called by the philosopher William Wimsatt as *functional localization fallacies* (59). For example, research in recent decades has focused on the impact of specific genetic mutations on cancer development and treatment response. While successful, this approach has also created blind spots, since environmental and biomechanical factors at higher scales are often ignored or held fixed as a methodological necessity. Genetics can infer a correlation of cancer treatment and treatment success. But this phenomenological association cannot be explained by genomics alone, as success of cancer treatment is also impacted by other systems such as the immune and endocrine system. Thus, the analysis of the cancer alone is insufficient, as it neglects the impact of the treatment on other parts of the organism.

### Level of Conceptual Resolution

It is often assumed that the precision of models increases as more details are incorporated, and some have even argued that the principle of Occam's razor does not apply to biology in the computational age (60). Inclusion of ever more molecular details could impede the predictive capacity of models, especially if these are not contextualized within the overall system organization: Living systems exhibit what Mihajlo Mesarovic termed *bounded autonomy of levels* (61). Cross-level relations in biology are neither independent nor linearly coupled—rather, they are dynamically autonomous within certain boundaries. Examples are how phenotypic states are often resilient to genetic mutations or changes in expression levels. As living systems are not homogenously organized, upscaling of models is not accomplished by simple averaging of lower-scale details. In contrast, multi-scale modeling of living systems requires an understanding of how the system is hierarchically organized, and how higher-scale structures can exert *top-down control* over lower scales through constraining relations (62). An example which is more elaborated below is stress-dependent release of cortisol with downstream effects on organ-level (63).

### Top-Down Causation

The relevance of top-down influences is exemplified by recent insights from multi-scale modeling of the human heart and the cardiovascular system (41), embryonic development (64), fracture risk in bone (42), organogenesis, and cancer (65, 66). In these contexts, macro- and meso-scale models represent higher-level features that act as *boundary conditions* for models at lower scales (67). Top-down causation occurs when higher-level structures shape lower-level interactions and channel dynamic possibilities, some of which would be impossible to reach for an unconstrained system (62). Just like the heart rhythm is possible due to constraints of the cell membrane and higher-level structures (37), the wiring of biological networks constrains lower-levels states and give rise to generic functions such as feedback control or signal implication. System theory can help to identify similarities in the patterns of organization in different systems, and hence to recontextualize the inputs from data-intensive fields (40).

## Organismal Systems Medicine as an Integrative Framework

As already mentioned briefly, some of these theoretical challenges for systemic thinking were already tackled by the philosopher and biologist Ludwig von Bertalanffy who made significant contributions to the field through his formulation of General System Theory (54). This theory should enable researchers from different disciplines to conceive their epistemic object as a dynamic system. Although his conception was already grounded on early biochemistry, molecular biology, and mathematics, he proposed an organism-centered view (organismic systems biology) (53). He underlined this idea by defining a system as a “structured whole.” In addition, the perspective of developmental biology was crucial for his concept of a theoretical function-oriented biology.

### Concepts and Models

Ludwig von Bertalanffy, Mihajlo Mesarovic, and other founders of systems biology, like James G. Miller, proposed explicit *conceptual multi-level models* to describe, explain, and predict dynamics of states of living systems. These ideas had also interdisciplinary relevance as GST was also developed in context of sociology by Talcott Parsons who designed a heuristic scheme that assumes that social systems have to fulfill four basic functions: adaptation, goal attainment, latent pattern maintenance, and integration (68). In consequence, these concepts could probably be useful for a general functional understanding of the organism and its pathology.

One of the most significant basic and already elaborated concepts is the notion of “dynamic equilibrium” (German notion: *Fliessgleichgewicht*) that governs processes on different levels of the organism. It is constituted by *asymmetric antagonistic convergence operations* of systemic cellular and molecular components, e.g., the nervous system (“autonomic network” of ergotropic sympathetic vs. trophotropic parasympathetic autonomous nervous system) (69), endocrine system (blood glucose regulation by antagonistic hormones) and immune system (pro- vs. anti-inflammatory agents). The action of these subsystems converge overlapping on sites of homeostasis of organ functions such as cerebral stress reactions, glucose homeostasis or balanced defense reaction. This concept of a dynamic interplay between *accelerators* and *brakes* can be a guiding principle not only to describe, but also to explain and understand temporal patterns of organismic processes in health and disease.

### Systemic Methods

**Figure 2:**
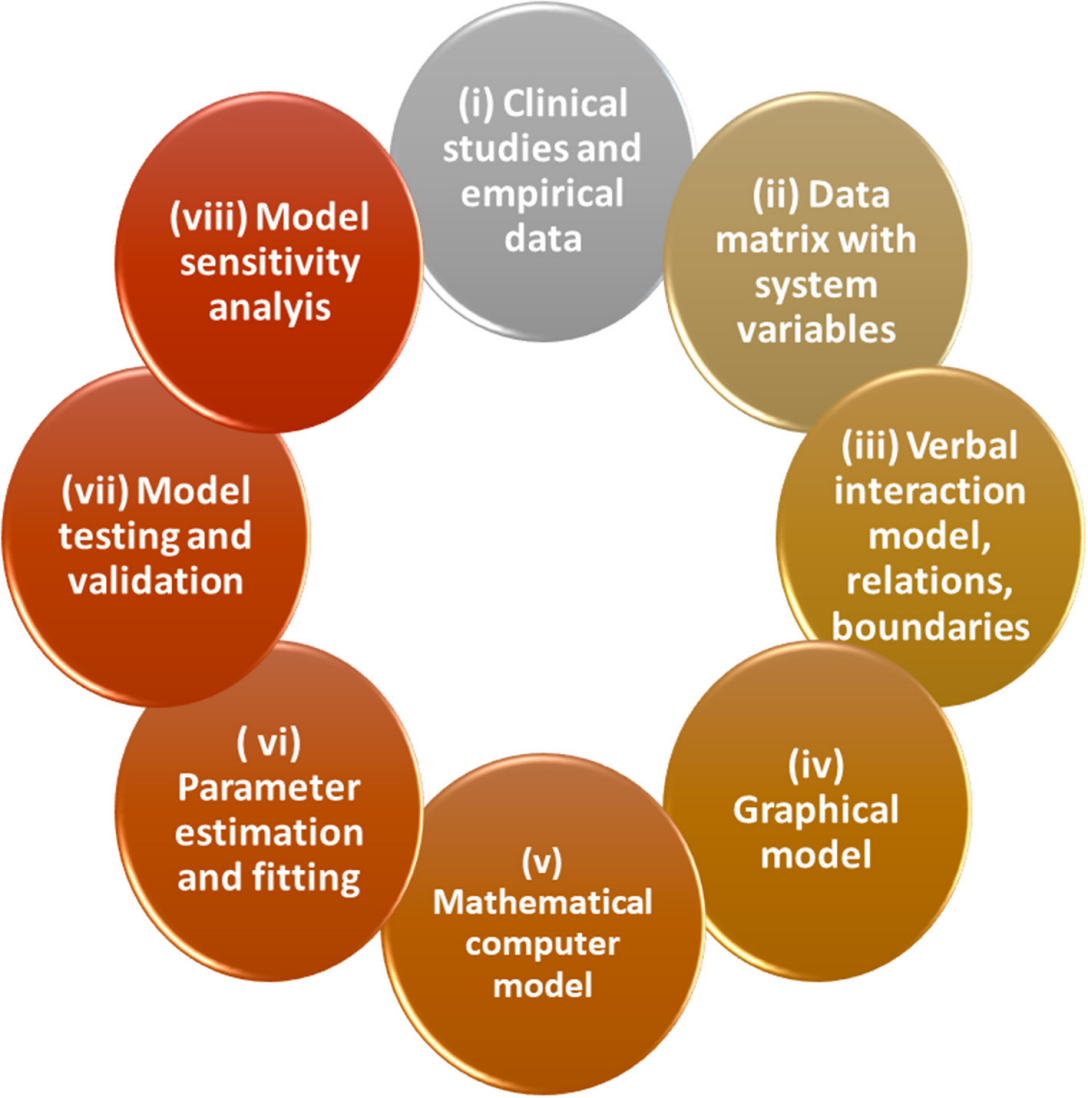
Methodology for iterative optimization of exploratory and quantitative models in Systems Medicine. In the following the workflow is described: (i) data collection, observations in a “transdisciplinary” groups, (ii) generation of data matrices, (iii) drafting a verbal model of interactions of components, definition of the system, elements, relations, and boundaries necessary for the subsequent formal models (iv) construct graphical model of causal loops and/or of stocks and flows/effects (decide about graphical language), (v) drafting mathematical equations, (vi) parameter estimation and fitting using existing data, state and flow variables, and coefficients (if not possible: “educated guess” by expert-based estimations), (vii) transposition to computational model simulation, model tests, validation, scenarios: “…if, then…,” (viii) model prediction and extrapolation and comparison with data, further validation and model optimization with backward improvements (for further details see text).

**Figure 3:**
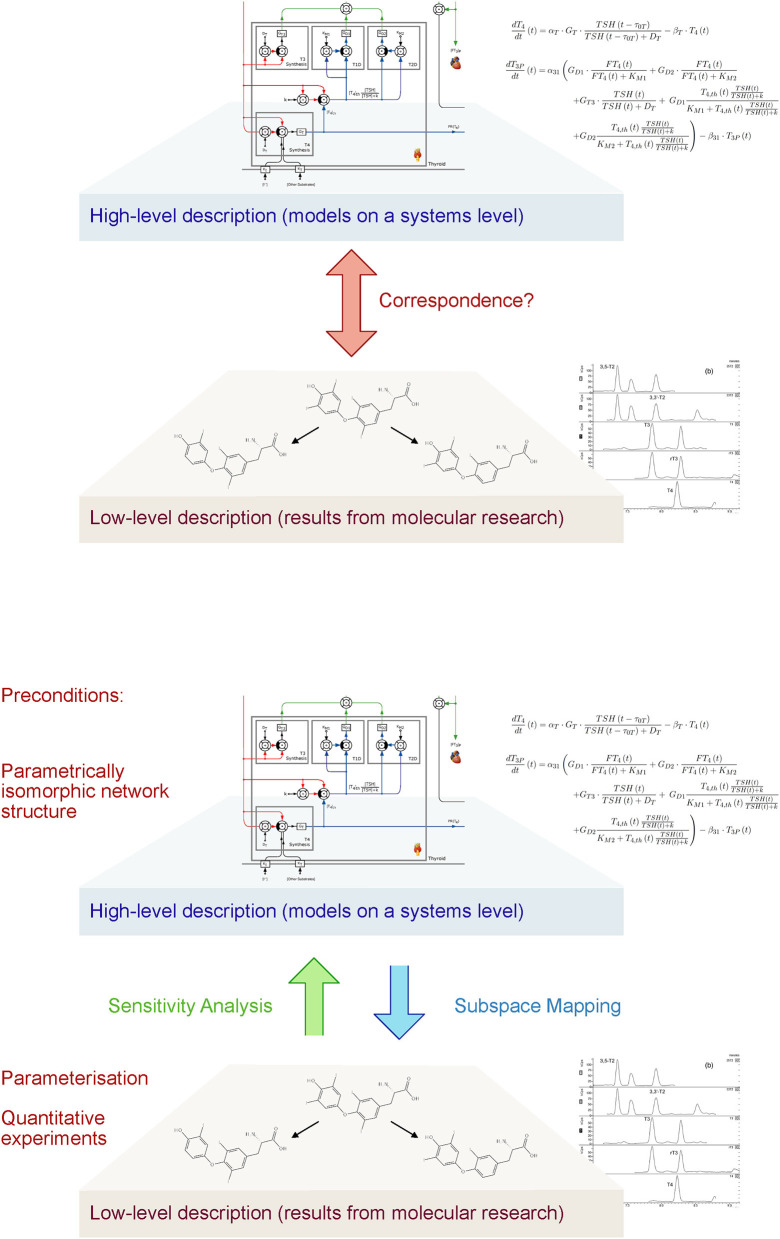
Today's biomedical research is faced by the challenge that descriptions of the systems of interest are restricted to different sublevels. A high-level theory covers a systems view, addressing large subsystems, e.g., multi-organ feedback control systems or even the whole organism including psychosocial relations. This is complemented by a low-level description of molecular structure and reactions. Unfortunately, the vertical translation, e.g., how molecular data are integrated into the high-level system description, is difficult. Methods of vertical integration include affine subspace mapping (top-down inference), sensitivity analysis (bottom-up reasoning) and graphical tools. They require, however, certain preparative steps on both tiers of research to be feasible (74).

During the last decades, several *methods of systemic modeling* were developed from qualitative models in context of systems dynamics, via mathematical models, computerized modeling tools, and data-driven inverse modeling (32). Aiming to construct comprehensive models with high ecological validity, transdisciplinary approaches that connect practitioners and researchers from various fields of relevance are the basis of successful modeling as it was elaborated in sustainability science (70, 71). Qualitative conceptual models are developed, formulated by simple verbal, graphical and tabulation tools (Figure 2). In a next step, the model will be transposed into a data-based mathematical formulation that can be used for exploratory computer simulations that need not much mathematical literacy such as programs Stella or Vensim (55). Notably, in this procedure the conceptualization of a system model can easily be done by graphical tools that facilitate interdisciplinary communication (Figures 2, 3). Already simple graphs of process structures, such as feedback and feedforward loops, can capture features of systems dynamics. Complex structures of process conditions (e.g., biochemical pathways) can be studied in this view qualitatively by identification of generic dynamic principles or “motifs” (72). This type of modeling is a classical forward approach, however, inverse or reverse approaches feeding data directly into model building are even more promising (19, 32). Data driven medical diagnostics and anamnesis are inverse problems *per se*. A classic example of biomedical inverse problems are imaging techniques from tomography to microscopy. Relating the acquired data to the unknown object is an inverse problem and requires mathematical modeling (73).

## Systems Pathology

Pathology is a key discipline in medicine and nowadays its subject is split into subsystems (organs and tissues) which are well-understood on the anatomical, physiological, cellular, molecular, and biochemical level. In line with our proposition for the utility of a function-oriented structural analysis, physiology, anatomy, and histology are central to make sense of the richness of these molecular data. Morphological macro- and micro-structures provide the boundaries and connecting structures that enable functions such as metabolism, respiration, circulation etc. The central challenge is to develop an integrative multi-level “systems pathology” that connects classical physiological and clinical knowledge with current rich molecular biological knowledge. The linkage between molecular and cellular models (bottom up) and the organ and organism level (top down) as in top-down causation models is a rather underrepresented approach (Figure 3). This step requires systematic methods of vertical integration of model inputs, translating from the molecular to the whole-system level and vice versa. One elaborated and validated example is the integrative multi-level model of the heart activity covering molecular and cellular mechanisms (75) and also a multi-organ model of the thyroid control loops (76). Regarding COVID-19 only a few attempts were made to design a multi-stage systems view on the pathology of this disease (77, 78). Such models should be constructed basically as multi-level models that integrate organismal physiology with molecular studies. Methods of sensitivity analysis can be used in order to study the effects of molecular properties and events (e.g., enzyme kinetics, receptor binding and certain genetic variants) on the behavior of the whole system from cells, tissue, and organs up to the level of the organism. Conversely, “reverse sensitivity analysis” allows for concluding from the behavior on the organismic tier to an affine subspace of sensible parameter values on the molecular level (Figure 3).

Preconditions for this advanced modeling technique include certain measures on both the systems and the molecular levels. On the higher level, it is required that the network structure follows a “parametrically isomorphic” paradigm, i.e., that the model is constructed from building bricks that can be mapped to knowledge from molecular research in a bijective manner. On the other hand, research on the lower level has to deliver quantitative results providing meaningful information on stimulus-reaction relations, temporal dynamics etc., and thereby enabling parameterization of the high-level models (Figure 3). Reusable libraries of universal motifs and building bricks (e.g., feedback loops, feedforward motifs, antagonistic, and redundancy) helps to speed up and simplify the modeling process (79–81).

## Multi-Level/Multi-Function Pathology Integrating Neurophysiology, Endocrinology, and Immune System

Even on a semi-quantitative level, systemic modeling is heuristically relevant. Regardless of the details available on subtypes of cells, receptors, signaling molecules etc., there is lot of evidence of the antagonistic organization and regulation of an integrative neurophysiological, endocrine, and immune system. The integration of these subsystems has implications for neurophysiological complications, excess mortality rates, and polypharmacological treatment for COVID-19 (77, 82–84) but also the assessment of potential novel vaccines such as the mRNA vaccines and their short- and long-term adverse effects due to mass vaccination, e.g., autoimmune reactions and neurophysiological manifestations (82, 85–88). The interaction of the neurophysiological, endocrine, and immune system is discussed in the following.

### The Neurochemical Antagonism

Most clinical observations in neurology and psychiatry confirm the view that a delicate dynamic equilibrium exists between activating and inhibiting neurotransmitter systems, although anatomic and pharmacological details (e.g., receptor subtypes) are more complicated (55): activating (ergotropic) noradrenaline (NA) and inhibiting (trophotropic) acetylcholine (ACh) oppose partially to each other, with different patterns of dynamics. They represent a body-wide antagonistic regulation of functions (heart, blood vessels, lungs, pancreas, gut system, etc.). On a second operational level, synergistically connected with NA, the neurotransmitter dopamine (DA), and serotonin (5 hydrotryptamine, 5-HT) exhibit a partial antagonism. On the side of ACh, fast operating excitatory glutamate (Glu) and inhibiting GABA show also partial antagonism. Clinically, these interactions can be seen in disorders like Parkinson's disease with a dominance of acetylcholine over dopamine because of loss of dopamine cells: substitution of dopamine can induce a psychotic syndrome and in turn neuroleptic treatment of psychoses can evoke a Parkinson's syndrome. By integrating such antagonistic effects into a neurochemical network model that can be simplified as a “neurochemical mobile,” the neurochemical basis of several neuropsychiatric syndromes can be described, and explained even quantitatively by computer simulations (89, 90). Effects of new medications (glutamate antagonists) can be predicted as well (anti-depressive effects): in depression NA, DA, and 5HT exert a hypofunction in neurotransmission, compared to a hyperfunction of ACh, Glu, and GABA. In consequence, selective serotonin reuptake inhibitors (SSRIs) that enhance 5HT transmission work as antidepressants and also glutamate antagonists such as ketamine can reduce depressive syndromes (91). Interestingly, all these neurotransmitters operate on probably all organismic cells and many body cells even produce transmitters (e.g., immune system).

### The Endocrine System

The endocrine system is a multi-organ system partially centered around the pituitary gland (92). The principle of asymmetric antagonistic convergence is only weakly confirmed in the endocrine system, but at the peripheral organ level the interplay of glucagon and insulin confirm this concept. The most important hormone is cortisol, a steroid hormone produced in the adrenal glands. It involves a range of processes related to metabolism, stress and immune response. It works ergotropic and is partially synergistically to NA and classic thyroid hormones. The main feature of the cortisol system is its multi-level upstream connection with the CNS via hypothalamus and the pituitary gland. This connectivity is often quoted as hypothalamus-pituitary-adrenal or HPA axis: HPA axis elevates cortisol level which in turn inhibits the HPA axis via glucocorticoid receptors in hypothalamus and pituitary gland as a feedback inhibition. This feedback loop exhibits several cyber-systemic features that make several pathologies [e.g., stress; (63)] understandable: delayed feedback with consecutive oscillations, delays, adaptation, allostasis etc. characterize the dynamics of endocrine systems.

**Figure 4:**
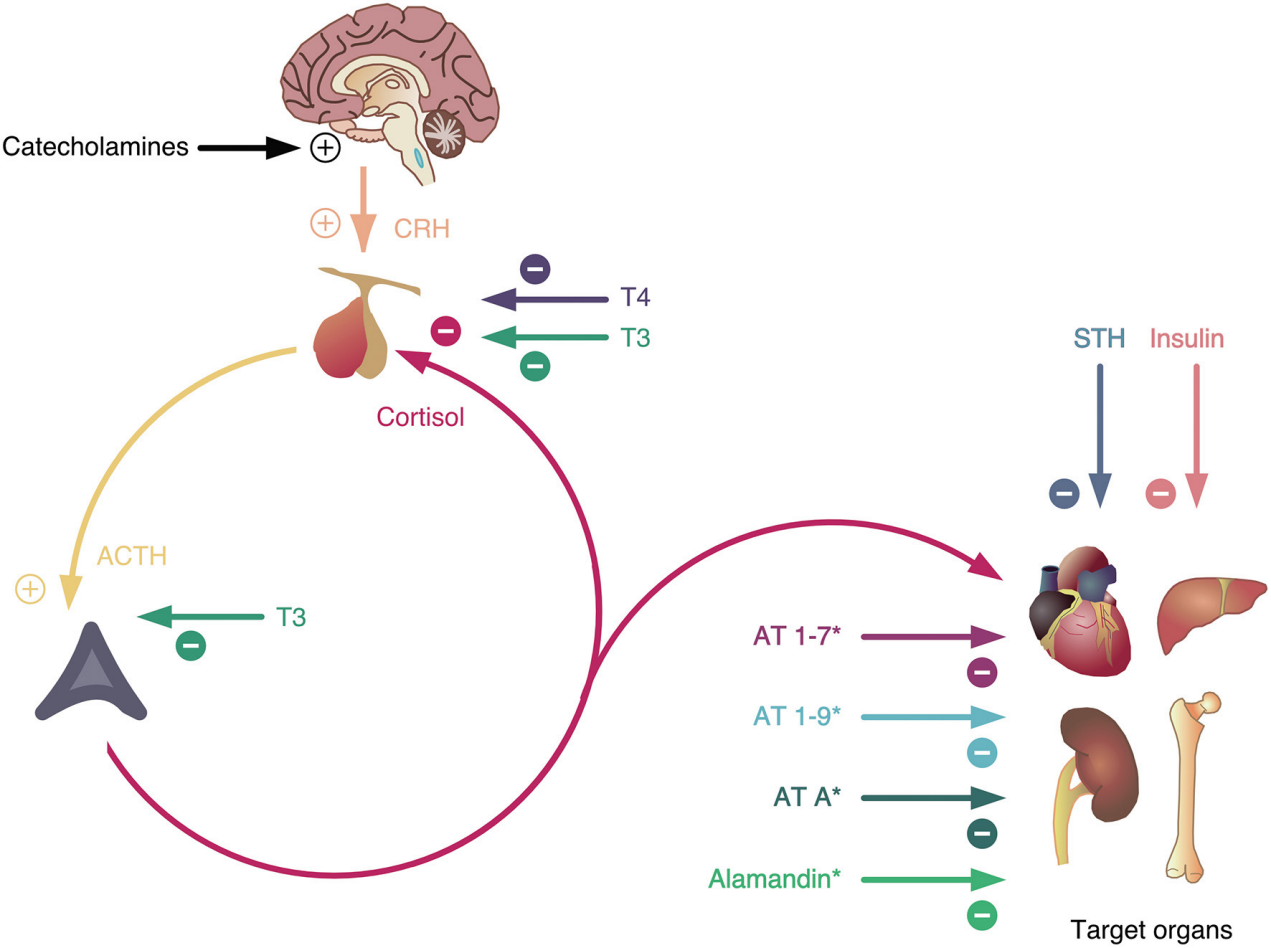
The hypothalamus-pituitary-adrenal (HPA) axis is the major stress mediator system on an intermediate time scale. It is stimulated by catecholamines (representing the fast stress system) and inhibited by thyroid hormones (as slow mediators of stress and allostatic load). In addition, it has multiple antagonists inhibiting partial functions at peripheral levels of the processing structure, some of them (marked by *) resulting from ACE2 activation. AT, angiotensin; STH, somatotropic hormone. For more details see text.

Interestingly, the HPA axis has multiple antagonists on various anatomical levels (each of them antagonizing certain partial functions) (Figure 4). They include growth hormone (anabolic action), insulin (glucose-lowering and anabolic function), hormones of the non-classical renin-angiotensin system (angiotensin 1-7, angiotensin 1-9, angiotensin A, and alamandine with hypotensive and hyponatremic actions) and thyroid hormones (HPT axis, central antagonism). It is therefore not surprising that the HPA axis is upregulated in critical infectious diseases, including COVID-19, while the HPT axis is downregulated (93).

### The Immune System

Regarding the principle of antagonistic convergence, the immune system has a (fast) *pro-inflammatory* and (slow) *anti-inflammatory* functional differentiation, for instance by signaling via Th1 and Th2 cells (94). Interferon (IFN) and tumor necrotic factor (TNF) are secreted from Th1 cells that amongst other effects activate macrophages and inhibit activity of Th2 cells that in turn can also inhibit Th1 cells by interleukin IL-4, IL-10 etc. In acute inflammation, Th1 subsystem dominates Th2 subsystem, in case of chronic inflammation Th2 subsystem dominates Th1 subsystem. In case of COVID-19, pro-inflammatory components exhibit a persistent overactivation. In response to a local pathogenic challenge, an innate immune response is initiated by type I interferons (IFN) and pro-inflammatory cytokines like tumor necrosis factor alpha (TNFalpha), interleukin 1beta (IL-1beta), and interleukin-6 (IL-6). Later on, when the adaptive immune response kicks in, an overreaction of the immune system is prevented by anti-inflammatory factors like TGF-beta and interleukin-10, thus, generating a negative feedback loop onto the immune response. In the case of COVID-19, this latter step sometimes fails to keep the immune response under control (84).

### The Multi-Level/Multi-Function Interaction

The complexity of these and other regulatory systems can be structured conceptually by a multi-level/multi-function interaction network. Regarding the immune system, it is well-known that ACh inhibits macrophages to secret TNF, whereas NA could stimulate TNF secretion via alpha- and beta-receptors (95). In synergy with ACh, cortisol also suppresses macrophage activity. Several other examples can be worked out (96), e.g., *multimorbitidy* and the problem of *polypharmacy* that affects about 20% of the population (97, 98). For instance, the comorbidity of diabetes mellitus and depression can be revisited by looking to molecular signaling cross-overs between the CNS (relative hypofunction of noradrenaline, dopamine and serotonin compared to acetylcholine, glutamate, and GABA in depression) and the physiological control of beta and alpha cells in pancreas physiology by these neurotransmitters and also the effects of insulin in the brain, etc. (99). In addition, imbalances within the immune system (elevated IL-6) contribute to the occurrence of depression (e.g., side effect of interferon therapy) and diabetes.

In consequence, these subsystem interactions need to be analyzed in detail on the basis of a reference network model. With regard to COVID-19, chronic bio-psycho-social stress situation could evoke the severe persisting shift in the immune system toward pro-inflammatory mechanisms.

## A Systems View on COVID-19—Integration of Epidemiology and Systems Pathology

The utility of systems thinking in medicine, especially in the case of COVID-19, is obvious in epidemiology by the universal application of SIR compartment models and their derivatives which help to understand and explore the dynamics of spreading of the virus (100). The diverse exposure features (asymptomatic carriers) are crucial for infection so that models have to be extended (2). At this population level, data analysis and modeling demonstrate the dangerous dynamics of exponential growth. Several theoretical challenges exist to represent the mechanisms of focal spreading and for evaluation of measures. They can only be partially solved by agent-based modeling, but only if the collateral effects of public health measures (home quarantine, lockdown) are also included in an ecological perspective of human beings (Figure 1). Systems theoretical analyses can help to explore and design management strategies ensuring health and economy, e.g., by cyclic management of lock down (101).

**Figure 5:**
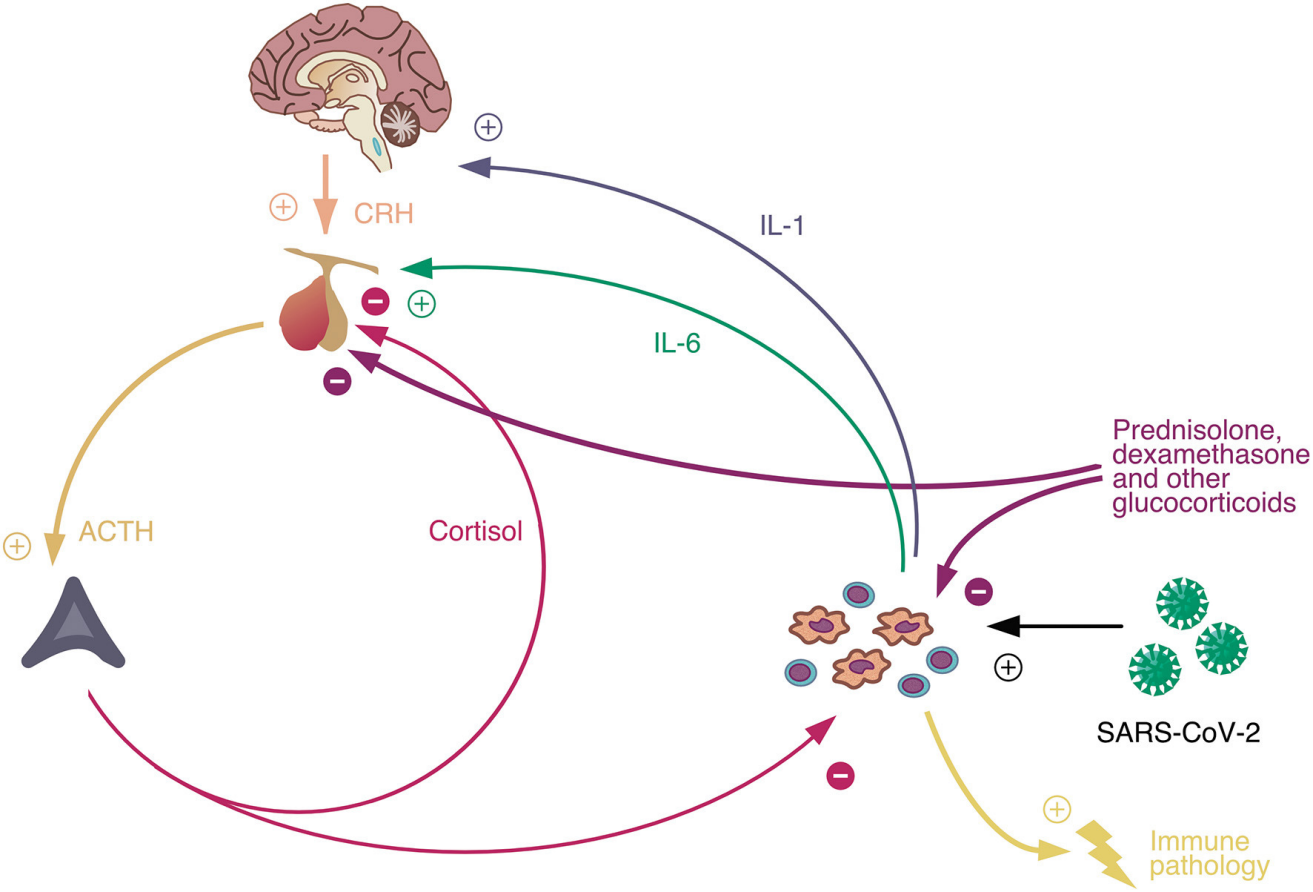
Interaction of the HPA axis with the immune system in COVID-19: Antagonistic convergence on ACTH production by inhibitory cortisol feedback and activating interleukin-6 that is released by macrophages after contact with SARS-Cov-2. The structure of the feedback loop explains the glucocorticoid paradox in COVID-19, i.e., that elevated serum concentrations of cortisol are associated with poor prognosis but that pharmacological use of glucocorticoids like prednisolone or dexamethasone leads to improved outcomes. For more details see text.

In addition, we propose an integrative compartment-based and multi-layer- and multi-level-oriented systems pathology, as a systemic understanding of Covid-19. It could help to explain the causes of asymptomatic clinical courses of SARS-CoV-2 infected persons. The complex pathophysiology of COVID-19 starts at the entrance of the virus in the compartment of the upper airways (nose, throat) with its local defense mechanisms on the layer of fluids that protect the mucosa (nasal mucus), the local expression of ACE2 on cells and the local presence of immune cells etc.. In this view, still the trivial pathophysiological question is not clarified if tonsillectomized individuals are at a higher risk that infection “jumps” down to the second compartment, the lower airways, respectively, to the alveola where the fatal mechanisms of hyper-inflammation occur: there might be a higher risk for respiratory dysfunction in tonsillectomized persons (102). Thus, the respiratory system in case of airborne virus invasion must be explored as a “structured whole” (compartment model), being connected with the circulatory system via alveoli thus providing oxygen for the whole organism and emitting carbon dioxide. In addition, each compartment should be conceived as being composed by a heterogeneous multi-layer tissue and should be modeled from tissue to cells to molecular processes of viral pathology addressed by molecular systems biological tools (103, 104). Here, one should not look only to effects on and of the molecular mechanisms of the *endocrine system* (renin-angiotensin system vs. cortisol system) in both directions but also consider the molecular effects on and of the *autonomous nervous system* (Figure 5). The crucial clinical problem of Covid-19 is that it appears as a *hyper-inflammatory process* as a result of a dynamic imbalance between pro-inflammatory and anti-inflammatory components of the immune system: On the immune systems level a macrophage and Il-6 excess is often reported that seems to lead to severe courses of Covid-19 (105). Also, a very high cortisol level is observed at hospital admission that could be functionally understood as an ineffective counterreaction, maybe because of down-regulation or desensitization of glucocorticoid receptors in macrophages (106). However, at first, the cellular invasion of the virus is based on utilization of the ACE2 receptor with the consequence that a lower amount of ACE2 is available that converts angiotensin II to angiotensin 1-7 and angiotensin 1-5 attaching to the mas receptor and operating antagonistically to proinflammatory ATR1 (107, 108). In consequence, the anti-inflammatory effects compared to proinflammatory effects are persistently lower than under normal conditions. This imbalance could explain the heavy structural and functional changes in the alveola. A next step might be the modeling of tissue dynamics in inflammation that can be explored by computer simulations of models of cell-cell interactions, namely as it was demonstrated for macrophages and fibroblasts showing that interaction structures based on growth factors can reach bistable homeostasis. This system theoretical concept that assumes a strong attractor basin in pro-inflammatory state space facilitates to understand pathological locked-in states of cell systems as they are found in alveolar pathology in Covid-19. As a starting point for a systemic view on COVID-19, a simple model could integrate the activation of the HPA axis by the inflammatory response triggered by virus invasion, where cortisol has again an immunosuppressant effect (Figure 5). The interaction of the involved three feedback loops could explain both markedly deranged blood glucose levels in diabetics infected with the SARS-CoV-2 virus (109), especially in severe cases (110–112), and the apparent paradox that therapy with glucocorticoids is able to improve the outcome of COVID-19 (113), whereas patients with elevated cortisol concentration face a poor prognosis of the disease (114).

## Concluding Remarks

A system-theoretical framework can provide a more consistent picture for complex diseases like Covid-19, by bridging the current gaps in medical knowledge, especially enhancing clinical knowledge, and experience. Systems theory enables the integration of multiscale top down (organismal view) and bottom up (molecular systems medicine) approaches. We propose the following unsettled strategies in systems medicine: (i) integration of biochemically-based and physiology-related dynamic models considering adaptive dynamic equilibrium, antagonism, and synergism, (ii) developing models of human and human health in an socio-ecological context with consequences for health status, and (iii) extending methodology of systemic modeling, also qualitative, pre-formal conceptualization techniques for the implementation of system-theoretical thinking and modeling technologies in the medical curriculum.

## Data Availability Statement

The original contributions presented in the study are included in the article/supplementary material, further inquiries can be directed to the corresponding author/s.

## Author Contributions

FT and WW conceived the study. All authors contributed and wrote parts of the manuscript.

## Conflict of Interest

The authors declare that the research was conducted in the absence of any commercial or financial relationships that could be construed as a potential conflict of interest.
